# Implementing FDM 3D Printing Strategies Using Natural Fibers to Produce Biomass Composite

**DOI:** 10.3390/ma13184065

**Published:** 2020-09-13

**Authors:** Waleed Ahmed, Fady Alnajjar, Essam Zaneldin, Ali H. Al-Marzouqi, Munkhjargal Gochoo, Sumayya Khalid

**Affiliations:** 1 ERU and Mechanical Engineering Department, College of Engineering, United Arab Emirates University, Al Ain 15551, UAE; 2 Department of Computer Science and Software Engineering, College of Information Technology, United Arab Emirates University, Al Ain 15551, UAE; fady.alnajjar@uaeu.ac.ae (F.A.); mgochoo@uaeu.ac.ae (M.G.); sumayya.khalid@uaeu.ac.ae (S.K.); 3 RIKEN, Center for Brain Science (CBS), Nagoya 463-0003, Japan; 4 Department of Civil and Environmental Engineering, College of Engineering, United Arab Emirates University, Al Ain 15551, UAE; essamz@uaeu.ac.ae; 5 Department of Chemical and Petroleum Engineering, College of Engineering, United Arab Emirates University, Al Ain 15551, UAE; hassana@uaeu.ac.ae; 6 Department of Electrical Engineering, National Taipei University of Technology, Taipei 106, Taiwan

**Keywords:** natural fibers, biofilters, FDM, 3D Printing, mechanical properties

## Abstract

Current environmental concerns have led to a search of more environmentally friendly manufacturing methods; thus, natural fibers have gained attention in the 3D printing industry to be used as bio-filters along with thermoplastics. The utilization of natural fibers is very convenient as they are easily available, cost-effective, eco-friendly, and biodegradable. Using natural fibers rather than synthetic fibers in the production of the 3D printing filaments will reduce gas emissions associated with the production of the synthetic fibers that would add to the current pollution problem. As a matter of fact, natural fibers have a reinforcing effect on plastics. This review analyzes how the properties of the different polymers vary when natural fibers processed to produce filaments for 3D Printing are added. The results of using natural fibers for 3D Printing are presented in this study and appeared to be satisfactory, while a few studies have reported some issues.

## 1. Introduction

Three-dimensional (3D) printing is manufacturing a 3D object from a computer-aided design model by sequential addition of materials added one layer at a time. It is also named as additive manufacturing (AM) [1]. The first 3D printing method was patented in 1986 by Charles W. Hull [2], and it was then known as stereolithography. Earlier in the 1990s, 3D printing techniques were used only for the creation of functional or visual prototypes and were more often referred to as rapid prototyping [3]. Currently, the comprehensive 3D printing market is growing in a fast-paced manner and is expected to expand even more in the next few years. 3D printing is being applied innovatively in multiple areas, including biotechnology, energy, medical devices, and many more [4,5,6,7,8].

The reason behind this fast-paced growth is that objects can be designed digitally and manufactured precisely in a layer-by-layer manner with no molds, dies, or lithographic masks [9,10]. The technology is now being rapidly adopted in both industrial and household settings, because of its many advantages, such as suitability for small scale production, effortless part acquisition, limited waste, energy efficiency, and no need for expensive tools [11]. 3D printing offers automation and reproducibility to a great level. It allows the uninterrupted production of structures that can only be produced with much more effort using traditional subtractive manufacturing procedures [7,12]. 3D printing has a potential for providing prototypes, customer-specific designs, high structural complexity, and rapid on-demand fabrication of small production lines at affordable rates [13]. Therefore, it is regarded as the next revolution in manufacturing.

With 3D printing, it is possible to fabricate objects of complicated shapes and thickness, which may be inaccessible to the standard polymer manufacturing techniques [14,15,16]. The printing techniques can broadly be divided into four categories: (1) extrusion-based methods, such as fused deposition modeling (FDM) where layers of material are fused in a pattern to create a printed object, (2) particle fusion-based methods, such as selective laser sintering which uses a laser to sinter powdered material, aiming the laser automatically at points in space defined by a 3D CAD model, binding the material together to create a solid structure, (3) stereolithography (SLA), the production parts are printed in a layer by layer fashion using photochemical processes by which chemical reaction causes the formation of polymers, this is mostly used for the production of prototypes and patterns, (4) inkjet printing which prints by depositing liquid materials or solid suspensions, (5) digital light process (DLP) is similar to SLA as both cure liquid resin using light. The primary difference between the two technologies is that DLP uses a digital light projector screen. In contrast, the SLA uses a UV laser, (6) multi jet fusion (MJF) which builds functional parts from powder instead of using a laser to sinter the powder MJF uses an inkjet array to apply fusing agents to the bed of powder, and (7) electron beam melting (EBM), is a metal 3D printing technology that uses an electron beam controlled by electromagnetic coils to melt the metal powder [13,17,18]. Although some materials can be used for printing using different 3D technologies, the compositions of the printable material vary considerably [13]. Among the various 3D printing techniques, one of the most popular techniques is the fused filament fabrication (FFF) or extrusion-based method because it is simple, cost-effective, and does not require hazardous solvents or glues [19]. Also, the printing apparatus is small in size that can be accommodated on a tabletop [20]. In this technique, an object is built by selectively depositing melted material layer-by-layer along a pre-determined path. The materials used are thermoplastic polymers that come in a filament form. The following printing parameters are used for the extrusion-based technique: (1) extruder-related (nozzle diameter and filament width), (2) process-related (temperatures and speed of printing), and (3) structure-related (layer thickness and infill geometry).

Thermoplastics are being widely used in extrusion-based techniques, since they have a low impact on the environment, as they are recyclable and are available in a great variety of materials. However, polymers, such as polylactic acid (PLA) and acrylonitrile butadiene styrene (ABS) or nylon can be hazardous and not adequately environment-friendly, as volatile organic compounds and ultrafine aerosols may be generated during 3D printing [21]. However, less toxic 3D printing materials are forthcoming. To reduce safety risks and unpleasant odors associated with synthetic polymers, the industry is now more inclined toward natural polymers, which are environmentally friendly and renewable [22]. Research is now more focused on developing printable biopolymer composites with improved performance. Environmentally friendly and inexhaustible biobased materials are now being investigated. This includes cellulose derived from plants, biomass from marine, wood, and agricultural residuals, and other abundant renewable feedstocks, which are potential alternatives to fossil resources [23,24,25].

The factors affecting the cost of the printed objects are the cost of materials being used and the time taken to print. Some filaments are expensive as compared to other filaments and printers may pose restrictions in their usage. The cost of filaments can be reduced by the addition of economical filler materials, which may improve the flexural stiffness, mechanical properties, and stability after solidification. However, to achieve these benefits, a suitable chemical treatment to the fibers may be required and a suitable coupling agent for material formation may also be needed. Moreover, the use of fillers will assist in mitigating the environmental impact.

Natural fibers have recently been widely used as additives in extrusion-based filaments [26]. To produce a good-value natural fiber thermoplastic filament, the biofilter should be mixed with a polymeric matrix. This can be achieved through compounding using a co-rotating twin-screw extruder, which allows dispersive and distributive mixing [27]. The latter homogenizes additives evenly within the matrix, while the former eliminates additive clusters and is particularly relevant for natural fibers, as they tend to attract one another. Mechanical performance is improved by the chemical treatment of fibers, and it positively affects the load transfer capability of the biofilter-polymer interface [11]. Even though the use of fiber reinforcement appears feasible and promising, it has various challenges that need be overcome, such as the effect of fibers on the resolution, agglomerate formation, heterogeneous composite formation, blockage of printer heads, non-adhesion, and increased curing times [5].

The polymer matrices can be categorized as either biodegradable or non-biodegradable, or, based on their origin, as a virgin, recycled, or hybrid, respectively. Few reviews have been published. The review of Wang et al. [26] summarizes different materials used for 3D printing, their properties, and application in fields of biomedical, electronics, and aerospace engineering. Another review by Mazzanti et al. [11] reviews the mechanical properties of 3D printed objects of polymers containing natural fillers. This review aims to cover recent advancements in the FDM process of polymers with AM techniques to inculcate natural fibers as fillers, their fabrication strategies and parameters, and their effects on the mechanical properties of the resultant 3D-printed parts.

## 2. Article Structure

Literature articles included in this review were selected based on several themes. These themes are discussed in detail in Section 3, Section 4 and Section 5 of this paper. The article is divided into seven major sections. Section 1 gives a brief background on the basics of 3D printing techniques. Section 2 explains the layout of the different areas in the article. Section 3 overviews the different materials i.e., type of polymers that have been used in the production of composites in the selected literature under review, categorized as a virgin, recycled, or hybrid. The literature that has been reviewed is concerned with polymers filled with natural fibers or particles and processed through FDM techniques. Section 4 discusses the filaments used and their effect on the mechanical properties of 3D-printed samples. Section 5 discusses the failures and challenges encountered by studies in printing with natural fibers. The discussion, conclusions, and possible future developments are presented in Section 6 and Section 7 respectively.

## 3. Materials

The invention of multi-material printers allows controlling the material composition and properties and offers layered composite materials. The availability of various types of printing heads has helped to produce and print blended composites with variable features. Because of the multi-material printing capability, a variety of improvements can be seen in the mechanical properties. However, the manufacturing and processing operations are complex. Recent progress in composite 3D printers has resulted in the development of pre-blended materials with fillers such as nanoparticles, carbon nanotubes, and fibers to accomplish unique features and capabilities [5].

Polymers, in particular, have widely been used in the industry due to the ease of fabrication and accessibility. The 3D printing industry mainly uses polymers in forms such as reactive, liquid solutions, and thermoplastic melts [28,29]. The profits, combined with enhancements from fiber reinforcement, offer a satisfactory combination for future improvements in the AM technology. To further decrease the environmental risk, bio-based or natural polymers are now being preferred, which is generally produced from waste materials or natural elements. Bio-based or natural polymer hydrogels such as algimate, collagen, keratin have been used to prepare composites for 3D printing and have shown great potential.

**Figure 1:**
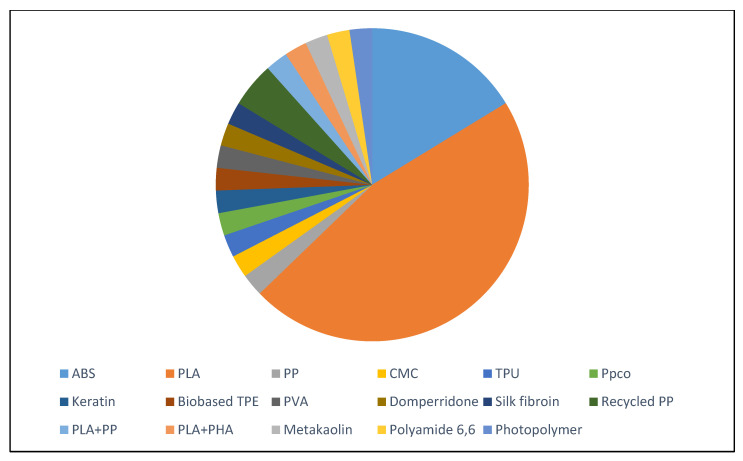
Different polymers used in reviewed studies.

The various polymers used in the selected articles reviewed in this paper are presented in Table 1 and Figure 1. Each sector represents the number of articles for a particular polymer. Out of 44 articles, 47% used PLA as their polymer with various natural fibers. It can be observed from Figure 1 that the ABS and PLA are the most commonly used polymers with natural fiber fillers. ABS virgin polymers are used widely as they are simple and easy to process, are readily available, have the required mechanical properties and toughness, have high melting strength, and durability. PLA is usually utilized for biodegradable plastics as it is readily available, environment friendly, and cost-effective, without compromising mechanical strength. It can easily be produced from lactic acid, which can be derived from the fermentation of cornstarch, sugarcane, or tapioca. Several materials, like polycaprolactone (PCL) or polyhydroxybutyrate (PHB), are also used. Many studies have used natural fiber, wood, or hemp as fillings for filaments. Tran et al. [19] prepared a biofilament using cocoa shell waste and PCL utilizing a single-screw extruder. The resulting 3D-printed specimens displayed a well-defined structure with good adhesion between deposition layers, and fine resolution. This material can potentially be used for household and biomedical applications.

This review divides the polymers into three categories, namely virgin, recycled, and hybrid. Virgin polymer is used in its original form while recycled polymer refers to the polymer sourced from the recycling of certain items. Hybrid polymers, on the other hand, is formed when two or more materials are combined. A total of 38 out of the 44 studies used polymers in their virgin form. Among virgin polymers, the most commonly used one was PLA (adopted in 17 studies) followed by ABS (utilized in seven studies). As shown in Table 1, other polymers include Carboxymethyl Cellulose (CMC), Thermoplastic Polyurethane (TPU), Polypropelene (PP), biobased Polyphenylene Ether (PPE), keratin, Polyprolactone (PCL), Polypropylene Copolymer (PPco), Polyvinyl Alcohol (PVA), Polyhydroxyalkanoate (PHA), bio based Thermoplastic Elastomer (TPE). These polymers were combined with natural fibers in varying proportions to investigate the fiber’s effects on the properties of printed objects.

To develop filaments for 3D Printing, various methods and strategies can be found in published studies. Filaments were created by using biomass fillers and polymers in different ratios, mixed or blended in various ways, and in some cases with the chemical treatment applied to achieve printer compatibility. The biomass fillers investigated in the reviewed studies were characterized by the parameters listed in Table 2. These parameters affected the mechanical and structural properties of the final printed objects.

**Figure 2:**
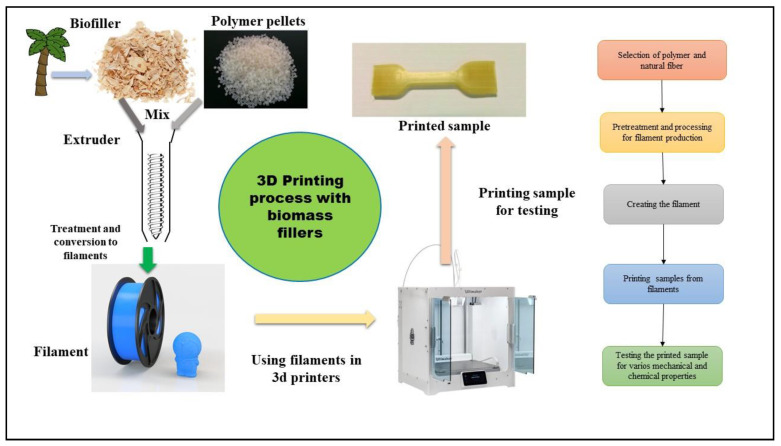
The cycle of 3D printing using natural fibers.

In most of the reviewed studies, the fillers were acquired in solid forms, such as pellets or fibers or their natural form, such as shells. They undergo various types of pretreatment before they could be used for filament production. These pretreatments involve sieving, crushing, grinding, washing with deionized water, adding certain chemicals to make them easier to blend, or drying in an oven to remove any moisture content which could hamper the product, before converting into the required form. Figure 2 shows the basic steps of 3D printing using natural fibers.

Most of the fibers were used in their powder form as shown in Table 2. However, few studies did use fibers in their original form as continuous fibers or in the yarn form dried and treated before being added to the polymer matrix. Once the fillers were ready to use, they were mixed with the polymer in varying quantities to generate filaments.

Fiber strengthening, in particular, is a good way to improve the properties of polymers. Pre-blended materials, using discontinuous fibers as an additive, have extensively been explored as a suitable filament substitute for multi-head printers with multifaceted and expensive designs. These materials display exceptional characteristics and capabilities, depending on the additive used. Mechanical, electrical, or thermal properties can reasonably be achieved. Natural fibers have recently been used as additives in FDM filaments. For a high-class natural-fiber-filled thermoplastic filament, the biofiller should be mixed with polymeric matrix and this can be achieved by compounding both the fibers and polymer, using a co-rotating twin-screw extruder, which allows a dispersive and distributive mixing. The latter distributes additives evenly within the matrix, while the former breaks additive clusters and is useful for natural fibers, as they attract one another.

## 4. Mechanical Properties

The mechanical properties of 3D printed objects are affected by structural and printing factors. These factors affect the internal structure of the object, which is strongly linked with the material in making the properties of the printed component. These may change substantially even if only a single parameter is modified. There are certain printing parameters that affect the mechanical properties (e.g., the nozzle diameter and type and printing temperature affects the structure and infill density). The amount of filler added affects unique properties of the printed object, such as its density and mechanical, flexural, tensile and structural properties. Several types of tests were commonly referred to in most of the reviewed articles such as the tensile and flexural tests. The fiber content in the filaments also affected the properties of the printed samples. It was observed that low fiber content gave positive results and, as the content increased, the properties reduced.

**Figure 3:**
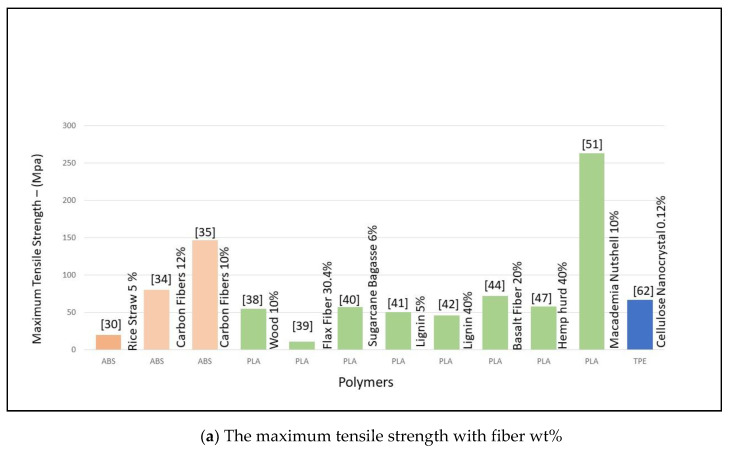

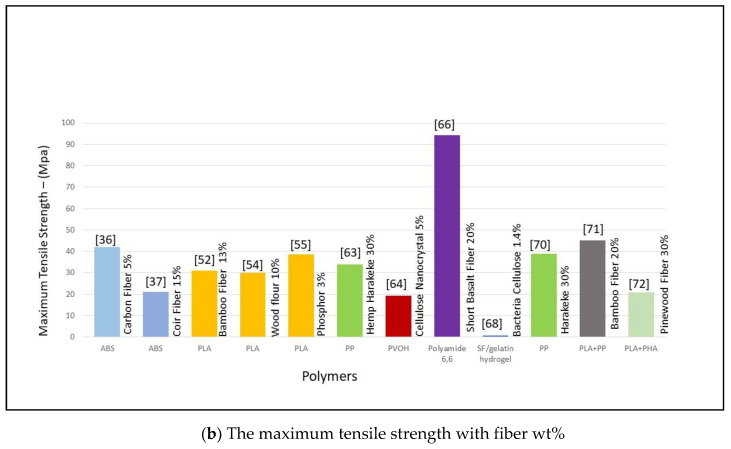
(**a**) The maximum tensile strength with fiber wt.% [30,34,35,38,39,40,41,42,44,47,51,62]. (**b**) the maximum tensile strength with fiber wt.% [36,37,52,54,55,63,64,66,68,70,71,72].

The below Figure 3a,b gives a quantitative analysis of the maximum tensile strength seen in different studies and the weight percentage of the fiber content in these filaments.

Osman et al. [30] used ABS with rice straw (RS) and a single-screw extruder to investigate the mechanical properties at varying fiber contents of 5–15 wt%. Specimens were printed to test tensile, flexural, and water absorption. It was noted that tensile properties decreased at RS increased, flexural properties decreased with the increase of RS but increased at 15%, and water absorption increased with the increase in fiber. The resultant filament was cost-effective and could be used to produce cheap prototypes. Girdis et al. [31] used ABS with macadamia nut shells in a different ratio (19–29 wt%) along with a binding agent (MAH 3%). The resulting filament was tested in tension and compression, the results demonstrated that the printed sample was very similar to wood-polymer composite filaments, but it had a lower density, making it suitable for the fabrication of lightweight products. Ahmad et al. [32] adopted ABS with oil palm fiber from empty fruit bunches where the fiber content was set at 5 wt.%. The resultant filament was used to print specimens and the tensile and flexural strengths were investigated. The results showed that the tensile strength and modulus of elasticity increased but the flexural strength decreased [32]. Kariz et al. [38] used wood powder in varying amounts up to 50 wt.% with PLA. The different filaments showed that the tensile strength decreased with higher wood content. Duigou et al. [39] used continuous flax fiber with PLA. The resultant filament had an increased tensile modulus and strength values as compared to previously published studies. Nguyen et al. [34] used lignin at 40–60 wt.% with ABS and noticed an increase in stiffness and tensile strength when discontinuous carbon fibers at 4–16 wt% were added to lignin, it achieved enhanced mechanical stiffness and printing speed. It reduced the nylon crystallization, allowing excellent printability at a lower temperature without lignin degradation. Liu et al. [40] used cellulose extracted from sugarcane bagasse (SCB) with PLA and discovered that the tensile strength of printed objects was best at 6 wt.% of SCB, and flexural modulus constantly decreased with the increase in SCB content. Yang et al. [35] used continuous carbon fibers at 10 wt.% with ABS and the results indicated an increase in flexural and tensile strength, but also showed a deficient interlaminar shear strength and low interface performance. Huang et al. [68] utilized silk fibroin and gelatin hydrogel along with bacterial cellulose nanofibers. The study indicated that the tensile strength of the printed sample increased significantly with the addition of BCNFs to the bioink. Zander et al. [69] used recycled polypropylene (PP) with wood flour, cardboard paper, and wastepaper. The dynamic mechanical analysis showed that the addition of cellulose materials increased the strength modulus. With the addition of 10 wt.%. cellulose, a 38% increase in the elastic modulus was noticed but no significant improvement in tensile strength was observed for virgin PP. Recycled PP with hemp harakeke fiber, which was supposed to affect positively the strength and shrinkage, was utilized by Stoof et al. [70]. The reason for the reduced mechanical properties was the stress relaxation of the polymer during printing, which was conducted at a lower pressure compared to filament production.

Long et al. [71] used PLA and PP with bamboo fiber and added 5% maleic anhydride grafted polypropylene (MAPP), which impacted the mechanical properties by improving the tensile and flexural properties and strength. The improvement in the mechanical properties was attributed to the fact that irregular grooves and cracks induced by the modification of bamboo fibers facilitated the infiltration of polymer into the fibers because of the strong capillary effect. Agnoli et al. [73] used microalgal biomass with lignin in geo-based polymer metakaolin and alkaline activator. When hardened, this composition displayed mechanical properties comparable to the unfilled material and a microstructure with smaller pores. Lastly, a printing test was successfully performed with a larger printer to assess the viability of producing large-scale structures. PLA+PHA with pinewood fiber was used by Guessasamo et al. [72], and the result revealed a tendency for heat accumulation at high printing temperatures. However, there was very limited improvement in the tensile performance at these temperatures, making 220 °C an ideal choice for printing the wood-based filament.

Mechanical performance can also be improved by the chemical treatment of fibers, which positively affects the strength of the biofilter-polymer interface. A total of 20 out of the 44 studies used some form of treatment in the production of filaments. Xie et al. [33], for example, assessed the effect of two different plasticizers and their mixture in different proportions and found that adding 4 wt% of tributyl citrate enhanced both strength and elongation at the breaking point. Bi et al. [58] tested different modifiers added to wood-TPU (thermoplastic polyurethane) composites and found that it is possible to improve the interfacial linkage between the TPU and wood fibers using diphenyl methyl propane disocyanate (MDI) and compensate for unwarranted flexibility by adding EPDM grafted with maleic anhydride (EPDM-g-MAH) as a compatibilizer.

**Figure 4:**
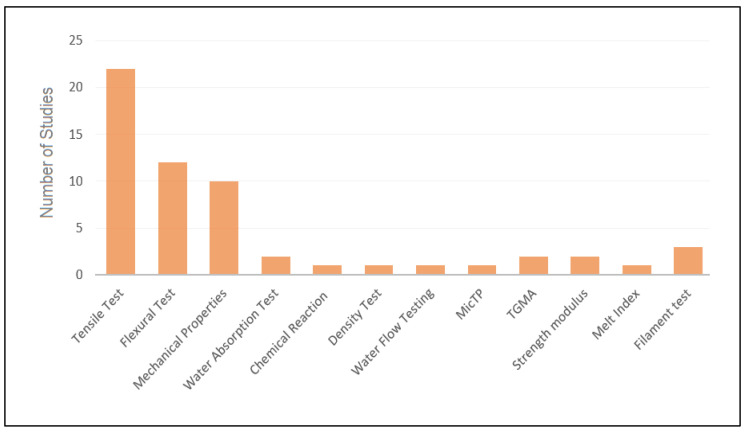
Types of tests conducted on the printed samples.

Figure 4 shows the frequency of different types of tests that were conducted throughout the studies. It can be noticed that tensile, flexural, and mechanical properties tests were the most commonly conducted tests. Under mechanical properties, the studies covered a variety of tests like water absorption test, porosity test, water flow test, strength modulus, chemical reaction test, filament tests, microtopography, and thermogravimetric analysis.

## 5. Printing Failures and Issues

There is no agreed standard or benchmark for the mechanical characteristics of 3D printed objects to regulate the tensile, flexural, and compressive properties of these objects. Also, the procession parameters play a significant role in identifying the mechanical properties, thus making it very difficult to draw a general conclusion when comparing the results of different studies. However, it was observed that, for ABS and PLA-based materials, natural fibers as fillers had a negative effect on the mechanical properties, i.e., when the filler content increases, the strength decreases, but there has hardly been any variation in stiffness compared to the unfilled material. It is worth mentioning that the majority of the studies listed above have incurred challenges and failures after printing the final object.

Osman et al. [30], for example, observed that the tensile strength decreased initially after the addition of rice straw to ABS, but increasing the rice straw content partially remediated the situation. Also, the flexural and modulus of the printed object decreased as the rice straw content increased. The water absorption also increased with an increase in the rice straw content due to increasing porosity, which in turn compromised the mechanical strength of the composite. Girdis et al. [31] found that increased macadamia nutshell content led to decreased density and strength in all samples of printed objects. Ahmad et al. [32] noticed that the oil palm fiber and ABS composite filament showed increased tensile strength, but the flexural strength decreased as the material was very brittle. Also, the microstructure of the composite showed that the fibers were not mixed well in ABS, as some were present in their insoluble form. Kariz et al. [38] concluded that an increased wood content in PLA resulted in a rough surface with increased voids and visible clusters of wood particles because of clustering and clogging of the printer nozzle. This higher amount of wood also decreased the storage modulus. Le Duigou et al. [39] noticed that the weakest point of their printed objects was the transverse properties that continued to stay lower than those of similar flax-PLA thermo-compressed composites. The damage mechanism observed during tensile tests was like that observed in continuous synthetic fiber-polymer printed composites with filaments unwinding.

Liu et al. [34] observed that the SEM analysis of fracture surface morphology of a 3D printed object revealed inner-line and interlayer voids. It was concluded that an increased content of the filler material reduced the tensile and flexural strength, and the increased porosity caused by higher mass fractions of the filler has a negative impact on the mechanical properties of the printed object. In the study conducted by Gkartzou et al. [41], it was found that the objects produced under the same conditions exhibited different fracture behaviors due to premature intra- and inter-laminar failure related to under- or over-extrusion or weak bondages between individual fibers. Tao et al. [43] noticed a decrease in the onset temperature of the thermal degradation of the composites, and the addition of 5 wt. percentage of wood flour had no effect on the melting temperature of PLA. Sang et al. [44] investigated the effects of adding the “KH550” treated basalt fiber to the PLA used for printing. They observed that the fiber length affected the mechanical properties even though the analysis showed that a longer fiber length improved the tensile and Young’s modulus. However, the flexural properties deteriorated with increasing fiber length, which produced large pores in the infill interlayer and led to adhesion failure in printed specimens. The experiments by Guen et al. [46] revealed that the mechanical properties were reduced by the addition of wood and rice husk fillers to the polymer due to the weakening of the inter-strand cohesion in the printed objects. Daver et al. [48] found that the tensile mechanical properties of the composites deteriorated as the cork content increased while the impact strength initially decreased with the introduction of cork but then increased as the cork content became higher. Viscoelastic properties, on the other hand, exhibited a decrease with increasing cork content in the composites.

Selvaraj et al. [61] showed that the additive had a very pronounced effect on materials. However, all objects exhibited inter-laminar shear failure. The absorption test of the objects with the additive showed high absorption rates as the additive on the surface of the filament tended to absorb more moisture. Tanase-Opedal et al. [42] found that lignin reduced the tensile property and strength, resulting in a lower quality printed part, however, adjusting the printing temperature counteracted the effect to some extent. Yang et al. [35] found that the interface performance was inferior and low inter-laminar shear strength was seen in the printed object. Xie et al. [33] concluded that the filaments, which were not treated with 4% tributyl-citrate (TBC), did not demonstrate good mechanical properties, compatibility, water absorption, or thermal stability. Ning et al. [36] observed that the porosity was the severest in specimens with 10 wt. % of carbon fiber and it was seen throughout the fracture interface, which in turn resulted in the smallest mean values of tensile strength, toughness, and ductility. In the experiments conducted by Safka et al. [37], it was observed that the presence of coir fibers had a negative effect on the printed objects, and they exhibited decreased mechanical properties, as fiber reduced the adhesion between layers. Similarly, Milosevic et al. [63] found that the composite printed material had inferior qualities compared to the filaments. Kearns et al. [53] noticed that the printed objects had weak binding between printed layers, though changing the heating and print bed was done to fix the issue. Zander et al. [69] analyzed the fractured surface of the printed object and found that failure was initiated at the interface and the interfacial strength was low. A study conducted by Stoof et al. [70] revealed that, even though the filament at 30% wt. of harakeke fiber had good tensile strength and Young’s modulus, the properties had reduced in printing. The reduction in mechanical properties was assumed to be due to the stress relaxation of the polymer during printing, as printing was conducted at a lower temperature compared to the temperature of filament production. Long et al. [71] compared injection molding-printed objects with FDM-printed objects and observed that the former had better properties, as in FDM each printed layer consisted of thermoplastic materials, which were deposited parallel to the printing surface resulting in lower bonding strength between the layers.

Guessasma et al. [72] noticed that higher temperatures above 230 °C were not advisable, as thermal degradation of wood particles occurred between 210 °C and 370 °C, and higher temperatures affected the tensile properties. It was concluded that the elongation of printed objects at the breaking point was fully restored and a loss of mechanical performance was seen by 41% and 35% stiffness and strength, respectively, using the best printing conditions.

## 6. Discussion

The 3D printing technique is one of the latest technologies, and most research efforts have focused on improving the quality of the printed objects by evaluating the mechanical and structural properties. Using natural fibers reduces the cost and is very beneficial for the environment, while the mechanical properties are not significantly affected by low filler contents. There have been numerous studies conducted in the past years evaluating properties and addressing challenges of biocomposite filaments using polymers with natural fibers in FDM technology. In this review, it was observed that, in the past five years, a significant number of studies had been carried out on natural fiber-based filaments to investigate the FDM and fiber effects on the mechanical properties of the final object. It was also noticed that, with regard to the properties of the final 3D printed object, there is no documented international standard to regulate the tensile, compressive, or flexural properties. The processing technique also influences the mechanical properties of the 3D printed objects, making it very difficult to draw a generalized conclusion when comparing different studies.

However, it appears that natural fibers, when added to ABS and PLA-based materials, had an undesirable effect on the mechanical properties. A decrease in strength was also observed by increasing the filler content. In other words, when less filler was used, the stiffness was the same as in the unfilled objects, but with an increase in the amount of filler, stiffness appeared to decrease. Remarkably, uncommon materials, such as PE and PP filaments with natural fiber fillers, seemed to exhibit improved properties due to their semi-crystalline nature.

In general, it was observed that, when biocomposites are utilized for 3D printing even though the stiffness is enhanced, the tensile strength hardly improves [66] and, in some cases, even deteriorates. Also, using short or discontinuous fibers in filaments tends to yield high porosity, which induces porosity in printed objects that are then most likely to absorb water making them not suitable for humid environments It was noted that the fiber content mostly varied between 5–30 wt% for maximum tensile strength, increase in fiber content reduced the tensile properties of the printed object. Above given Figure 3a,b captures the different maximum tensile strength achieved by studies along with the fiber %. However, there were many studies that did not give complete details of the fiber wt% or some didn’t mention the maximum tensile strength. Few studies did not include tensile testing in their evaluation of printed objects. Generally, the fiber content does not exceed 20–30% because the melting viscosity increases with an increase in concentration, thus, high power would be needed for extrusion through the nozzle. Also, the amount of polymer that can wet the fiber decreases resulting in a brittle filament. However, in the past three to four years, extensive research has been conducted and vast literature is available for studying the effect of formulation and processing with natural fillers and how they affect the mechanical properties of the printed product

The studies [19,31,32,33,43,45,46,48,53,57,58,60,61] were missing either one or both of information (fiber wt% or maximum tensile strength) and these studies [56,59,65,67,73] did not include tensile strength as a part of their evaluation.

When using natural fibers, the processing needs to be done diligently, or else it may lead to low-quality filament or poor outcomes. These fibers must be dried carefully in the initial phase even before compounding as it is very important to reduce the water content in them, which, if not done correctly, could lead to hydrolytic degradation. The temperature used should be monitored carefully to avoid thermo-oxidative degradation. If the material viscosity increases, it is important to have high extrusion temperatures. Still, high extrusion temperature also reduces the permanence time of the melt inside the heated chamber, which may prevent the degradation of the biofilters due to low heat conductivity in polymers. This needs to be handled by increasing the printing speed so that the permanence is reduced at very high temperatures.

Chemical treatments and toughening agents have proved useful for the improvement of tensile and flexural strengths. These agents may also tend to fill in the voids and cracks, reducing porosity and, thus, improving the strength. Some treatments are given before filament production to the fibers to improve their quality and make it easier to blend in with polymer for filament production.

It is also important to mention that nozzle type and size needs to be chosen with extra caution and understanding. A very narrow nozzle may lead to sieving of the filament during extrusion, which may lead to uneven material flow and may introduce defects in the printed sample. Similarly, a wide nozzle may tend to release more than the required amount of melted matter on to the plate, making it difficult to shape precisely, thus resulting in a very deformed and irregular printed sample.

Sometimes researchers tend to design their own custom nozzles, to meet a particular requirement of their protocol. For instance, Jassmi et al. [74] invented a compound nozzle for a cement 3D printer to produce thermally insulated composites, this nozzle can also be used with natural fibers to create insulation through the printing process.

Prior to printing, the filament quality must be assessed to check for any voids or cracks, and its composition should be cross-checked regarding the additives, their quantity, and distribution. For this assessment, various methods are used and scanning electron microscopy imaging is commonly used. This method can also be applied for verification and testing the accuracy of 3d printed samples.

The study by Liu et al. [75] proposed a novel method of 3D printing—the free-hanging 3D printing method for manufacturing CRFTP lattices. This method is different from the conventional layer-by-layer approach and uses direct extrusion of the overhanging and undercut structures with the guided spatial movement of nozzles. The method produced a better truss surface and bearing capacity, which negated the requirement of a complex support structure. However, this method is only suitable for continuous fiber material because the continuous fiber enables continuity and stiffness structure to the printed object [54]. In another study [76], TiO_2_ was used with ABS and extruded filaments to expand the chemical capabilities of the 3D printed structures, which were developed through thermoplastic printers. Zhang et al. [77] fabricated PLA with hydroxyapatite and compared the osteogenic and biodegradation property. Results showed that they had good osteogenic capability and biodegradation activity with no difference in inflammation reaction, showing the potential to be used in bone tissue engineering. Using 3D printing in bone tissue engineering with natural composites is gaining popularity. One study determined the practical setup of parameters to increase the properties of objects when using additives in powder form for tissue engineering [78]. Another study [79] created a cement-free 3D printed concrete by using desert sand, the cement was replaced by 10% silica fume and 30% fly ash. Along with a superplasticizer which was added in the range of 1 to 3%, by binder mass.

Even though some thermoplastics release hazardous gases when modified, when used as polymers with natural fibers as fillers, they tend to be less harmful to the environment. Non- biodegradable polymers like ABS can be recycled. Biodegradable polymers such as PLA can be safely degraded. The main advantage of using natural fibers as fillers is that the industrial waste or other discarded materials from factories can be put to efficient use in the creation of filaments, which in turn can be converted to newly printed objects. For instance, [54] uses wood powder waste collected from the furniture industry to create their filament with PLA. Many studies used jute fiber, flax fiber, or sugarcane bagasse for producing filaments in combination with different polymer, which in turn is giving a way to judicious waste management. To further reduce the environmental impact, researches are being conducted to produce printable biopolymer composites. Natural hydrogels, based on collagen, gelatin, and keratin, are being used to prepare scaffolds, which may be beneficial for tissue engineering. Industrial wastewaters and cellulose-based material are being put to good use, they are getting recycled into bio-based polymers and benefiting our environment in the long run.

Despite the importance of previous research efforts, there is some limitations. One limitation is that the fiber structure information was incomplete in many of these studies and average particle dimensions were generally used. Precise details would have enabled better understanding and determination of their performance as fillers. Many chemical agents were used as toughening agents and compatibilizers, which also affected the properties of printed objects. When used as additives, they created a multi-phased structure that reduced the concentration of stress and absorbed energy on impact. Several issues arise during printing, such as increased viscosity and fiber-matrix interface issues. Moreover, it has been observed that there is a lack in the studies related to the impact of implementing different internal 3D printing structures on the mechanical properties [80] of 3D printed fiber-reinforced composite as well as the influence of the open-source 3D printer [81] on the quality, consistency and the process capability [82] of the natural fiber 3D printed composite objects or using hybrid composite 3D printing technology enhanced with hard particles [83].

## 7. Conclusions

When natural fibers are added to ABS and PLA-based materials, less desirable mechanical properties of the FDM 3D printed products are observed at high biomass contents. For example, the strength properties decreased with increasing filler content. At low filler contents, the stiffness was the same as in the unfilled objects but with an increase in the amount of filler, stiffness decreased.

It is also important to note that the formulation of materials that can change the transfer of heat or flow properties would be most desired and should be reflected in future advancements in the field. Furthermore, careful analysis of the filament quality and composition before printing is important. Issues can be managed by choosing appropriate processing parameters, but FDM has a large number of variables and it is not easy to isolate the correlations between the structure and the properties. Also, this can be material dependent and also related to each other. Future investigations related to natural fiber-filled polymers are still required. Moreover, the effect of the factors may also be material dependent and interrelated among each other, thus, further examinations in this direction for natural fiber-filled 3D-printed polymers are absolutely required. More focus should be given in utilizing the industrial waste in the creation of bio-based polymers, to further reduce the impact on our environment.

## Figures and Tables

**Table 1:** Types of Polymers.

Polymer Type	Polymer Name	References
Virgin	PCL	[19]
	ABS	[30,31,32,33,34,35,36,37]
	PLA	[38,39,40,41,42,43,44,45,46,47,48,49,50,51,52,53,54,55,56]
	TPU	[57]
	CMC	[58]
	PPco	[59]
	Keratin	[60]
	Resin	[61]
	Biobased TPE	[62]
	PP	[63]
	PVA	[64]
	Domperidone	[65]
	Polyamide 6,6	[66]
	Photopolymer	[67]
Recycled	Silk fibroin (SF)/gelatin composite hydrogel scaffolds	[68]
	Recycled PP using cellulose waste materials	[69]
	Recycled PP	[70]
Hybrid	PLA + PP	[71]
	PLA + PHA	[72]
	Metakaolin, bentonite, and distilled water	[73]

**Table 2:** Biomass and Printing Parameters in Reviewed Studies.

Polymer Name	Biomass Name	Biomass Type	Biomass Size	Biomass %	Chemical Agent	Nozzle Diameter (mm)	Filament Diameter (mm)	Printing Temperature (°C)	Filament Process	Tests	Ref.
PCL	Cocoa shell waste	Ground	50 mm	0–50 wt%	-	0.5–0.9	100 200	-	LDM extruder	FT	[19]
ABS	Rice straw	Grounded	0.149 mm, 0.105 mm	0, 5, 10, 15, and 20 wt%	-	0.5	1.75	230	Single-screw extruder	T, F, A,	[30]
ABS	Macadamia nutshell	Grounded	Macrosize (MSZ)	19–29 wt%	-	1	0.3, 1.75, 6	250	Single-screw extruder	T, F, WFT	[31]
ABS	Oil palm fiber	Fibers	MSZ	5 wt%	-	0.5	2.5	210	Single shot extruder	T	[32]
PLA	Poplar wood flour	Powder form	MSZ	-	4% glycerol 2& glycerol 2% 4-tert-Butylcatechol	-	1.75	170	Twin-screw extruder	T, MI	[33]
ABS	Lignin and carbon fibers	Hot-pressed	MSZ	40–60 wt. % lignin 4–16 wt% carbon fibers	-	0.4	1.75	190	Twin-screw extruder	MP, MicTP	[34]
ABS	Carbon fiber	Fiber	Diameter of 7.2 mm	3, 5, 7.5, 10, 15 wt%	-	0.35	-	230	-	T, F	[35]
PLA	Poplar wood flour	Powder form	MSZ		glycerol tributyl citrate	0.4	1.75	220	Single-screw extruder	MP, MI	[36]
ABS	Coir fibers	Powder	MSZ	15 wt%	-	0.4	1.75	230–245	-	T	[37]
PLA	Wood	Powder form	0.237 mm	0–50 wt%	-	2	1.75	80–100	Single-screw extruder	T, ST	[38]
PLA	Continuous flax fiber	Yarn form	MSZ	-	-	-	1.75	140–165	Double screw extruder	T, SM	[39]
PLA	Sugarcane	Cellulose fiber	MSZ	3–15 wt%	-	0.2–0.4	1.75	80–100	Single-screw extruder	T	[40]
PLA	Pine lignin	Powder form	MSZ	5–20 wt%	-	0.4	1.75	200–210	Screw extruder	T, SM	[41]
PLA	Lignin	Liquid form	MSZ	0, 20, 40 wt%	-	1.75	1.75	230	Single-screw extruder	T, F	[42]
PLA	Wood flour	Powder form	MSZ	5 wt%	-	0.4	1.75	210	Single-screw extruder	T, F, SM	[43]
PLA	Basalt fiber and carbon fiber	Fiber form	1–3 mm	5–20 wt%	-	1.8	40.4	195	Flat-head nozzle	T	[44]
PLA	Grass biomass	-	MSZ	-	Pretreatment: 1 alkali- H_2_O_2_, 3% (*v*/*v*) H_2_O_2_, 1.5% (*w*/*v*) NaOH and 12.5 g/L Na_2_SiO_3_ 2. acid treatment: silvergrass was pretreated with 1.5% (*w*/*v*) of H_2_SO_4_ PLA was mixed with biomass and coupling agents	0.75	1.75	190–200	Co-rotating twin-screw extruder	MP, CR	[45]
PLA	Rice husks Wood flour	Both in powder form	MSZ	10 wt%	-	2.7	-	200	Co-rotating twin-screw extruder	MP, TGMA	[46]
PLA	Hemp hurd	Powder form	50 μm	-	Poly butylene adipate-co-terephthalate)(PBAT), ethylene-methyl acrylate-glycidyl methacrylate terpolymer (EGMA)	0.8	1.75	230	Single-screw extruder	T, DT	[47]
PLA	Cork	Powder form	MSZ	5 wt%	TBC	0.30	-	>130	Twin-screw extruder	MP	[48]
PLA	1.Wood 2. Ceramic 3. Copper 4. Aluminum 5. Carbon fiber	-	MSZ	-	-	0.4	1.75	200	-	T, F	[49]
PLA	Jute fiber Flax fiber	-	Jute fiber 2 mm Flax fiber 0.5 mm	-	-	0.2	-	215	-	T, F	[50]
PLA	Macadamia nutshell	Powder	MSZ	0, 5, 10, 15 wt%	Zirconium balls	0.4–0.6	1.75–0.3	210	Single-screw extruder	MP	[51]
PLA	Bamboo Flax	-	MSZ	15 wt%	-	-	2.85	-	-	FT	[52]
PLA	Cellulose fiber	-	MSZ	0–20 wt%	-	0.5	2.85	210	Two step extruder		[53]
PLA	Commercial grade wood powder waste	Powder	-	5–20 wt%	MAH NaOH	-	1.5 mm	-	Twin and single screw extruder	MT, T	[54]
PLA	Phosphor	Powder	500 μm	2 wt%	Toughening agent	1.75mm	1.75 + 0.05 mm	170–180	Singe screw extruder	T, F	[55]
PLA	Continuous flax fiber	Yarn	-	-	-	-	1.0 mm	190	-	Compressive strength	[56]
TPU	Poplar wood flour	Powder form	150 μm	10–40 wt.%	EPDM-g-MAH, POE-g-MAH, chitosan, MDI 5wt. %	0.4	1.45–1.75	180–200	-	FT, F	[57]
CMC	Natural cellulose	Fibers	100–200 μm	35–50 wt.%	Distilled water	0.4	1.75	210	-	T, ST, TGMA	[58]
PPco	Cellulose nano-fibers	Suspension form	MSZ	0–15 wt.%	MAPP	0.4	1.75	200	Single-screw extruder	MP, ST	[59]
Keratin	Lignin	Aqueous solution	MSZ	15, 20, 30 wt.%	polyethylene gly- col (PEG)	-	-	-	-	T, F, A	[60]
Elium^®^ liquid thermoplastic resin	Flax natural fiber	-	MSZ	5 to 15 wt.% of matrix	tamarind seed powder	0.8	-	230	Novel extruder	3PT Test, T	[61]
Biobased TPE	Cellulose nanocrystals	Spray dried	MSZ	-	-	0.4	-	178	-	T	[62]
PP	Hemp	Fiber	MSZ	10–30 wt.%	Alkaline	3	2.4–3.1	174–18	Twin-screw extruder	T, FFT	[63]
PVA	Cellulose nanocrystals	Microcrystals	MSZ	2–10 wt.%	-	0.35		230	Single-screw extruder	T	[64]
Domperidone	Hydroxypropyl Cellulose	-	MSZ	80–90 wt.%	-	0.2	1.76	210	Twin-screw extruder	MP	[65]
Polyamide 6,6	Short basalt fiber	Fiber	137 μm	20 wt.%	Portland cement	-	-	270–290	Tein screw extruder	T	[66]
Photopolymer	Abaca & Cabuya	-	-	20 wt.%	-	-	-	-	-	-	[67]
SF/gelatin composite hydrogel scaffolds	Bacteria cellulose nano-fibers	-	MSZ	1:2 ratio	-	0.3	1.77	-	-	MP	[68]
Recycled PP using cellulose waste materials	Wood flour Cardboard Wastepaper	Powder form	MSZ	5, 10, 20 wt.%	-	0.8	2.2	220	Twin-screw extruder	T	[69]
Recycled PP	Hemp+harakeke	Fiber	MSZ	10–50 wt.%	Alkaline	1	3	230	-	T, F	[70]
PLA + PP	Bamboo fiber	Dried fiber	MSZ	20 wt.%	MAPP	-	-	150–170	Co-rotating twin-screw extruder	MP	[71]
Metakaolin, bentonite, and distilled water	Microalgal biomass species and lignin	Freeze-dried powders	MSZ	1, 3, 5 wt.%	Bentonite	2.25	-	-	Piston-type extruder	ST	[71]
PLA+PHA	Pinewood fiber	-	MSZ	30 wt.%	-	0.4	1.75	210–250	-	T	[73]

MSZ—Macrosize, A—absorption testing, T—tensile testing, C—chemical testing, F—flexural testing, FT—filament testing, MP—mechanical properties, CR—chemical reaction, DT—density testing, WFT—water flow testing, MicTP—microtopography, TGMA—thermogravitometric analysis, SM—strength modulus, MI—melt index.
